# Draft Genomic Sequences of Streptomyces misionensis ACT66 and Streptomyces albidoflavus ACT77, Bacteria with Potential Application for Phytopathogen Biocontrol

**DOI:** 10.1128/MRA.00949-19

**Published:** 2019-09-05

**Authors:** Victor Satler Pylro, Armando Cavalcante Franco Dias, Fernando Dini Andreote, Alessandro de Mello Varani, Cristiane Cipolla Fasanella Andreote, Iron Amoreli de Figueiredo Ribeiro, Isabella Takahashi Kitano, Eduardo Roberto de Almeida Bernardo

**Affiliations:** a Department of Biology, Federal University of Lavras (UFLA), Lavras, Minas Gerais, Brazil; b Andrios Assessoria, Piracicaba, São Paulo, Brazil; c Soil Microbiology Laboratory, Luiz de Queiroz College of Agriculture, University of São Paulo (ESALQ/USP), Piracicaba, São Paulo, Brazil; d Departamento de Tecnologia, Faculdade de Ciências Agrárias e Veterinárias, Universidade Estadual Paulista (Unesp), Jaboticabal, São Paulo, Brazil; e Agrivalle Brasil Indústria e Comércio de Produtos Agrícolas, Ltda, Salto, São Paulo, Brazil; Georgia Institute of Technology

## Abstract

Here, we report the draft genomic sequences and annotation of Streptomyces misionensis ACT66 and Streptomyces albidoflavus ACT77, which are two bacteria with potential application for phytopathogen biocontrol.

## ANNOUNCEMENT

Phytopathogen biocontrol using bacteria is a promising and eco-friendly strategy for replacing or reducing the application of agrochemicals (1). The knowledge of genomic features may help us to understand the mechanisms involved in the interaction between biological control agents and the target phytopathogen, which is crucial for enhancing the use of these organisms in agriculture. Streptomyces misionensis strain ACT66 and Streptomyces albidoflavus strain ACT77 are Gram-positive bacteria commonly found in soil, as are the other Actinobacteria. Preliminary analyses have indicated that these isolates show *in vitro* fungicidal activity, suggesting their potential application for phytopathogen biocontrol *in situ* (data not shown). To gain insight into the use of these bacteria for biological control of phytopathogens, we performed whole-genome sequencing (WGS). Several *Streptomyces* spp. have been used to promote plant growth and for phytopathogen biocontrol, for example, against Magnaporthe oryzae in rice (2), Fusarium spp. (3), and some wood decay fungi (WDF) (4). *S. misionensis* strain ACT66 and *S. albidoflavus* strain ACT77 were isolated from soil under agriculture management in Brazil, by the dilution plating technique onto 5% tryptone soy agar (TSA; Bacto BD, USA) supplemented with 50 mg ml^−1^ of benomyl. The plates were incubated at 28°C, and the isolates were kept as pure cultures. To perform the WGS, the isolates were cultivated in nutrient agar for 48 h, at 28°C, and the genomic DNA was extracted using the Wizard genomic DNA purification kit (Promega) following the manufacturer’s instructions.

Paired-end sequencing libraries (2 × 250 bp) were constructed using the Nextera XT kit (Illumina, San Diego, CA) following the manufacturer’s instructions and sequenced using the Illumina MiSeq platform (Illumina). After quality filtering using Trimmomatic version 0.33 (5) (parameters [paired-end reads] included trailing, 10; leading, 10; slidingwindow, 4:10), a total of 1,185,642 paired-end reads were obtained for *S. misionensis* strain ACT66, and 540,058 paired-end reads were obtained for *S. albidoflavus* strain ACT77, consisting of a genome coverage of ∼70× and ∼36×, respectively. All reads were reference-based assembled with SPAdes version 3.12 (6), using *S. misionensis* strain DSM 40306 (GenBank accession number NZ_FNTD00000000) for *S. misionensis* ACT66 and *S. albidoflavus* strain NRRL B-1271 (NZ_JOII00000000) for *S. albidoflavus* strain ACT77 as references. The obtained contigs were further processed with the SIS software (7) to generate a set of contig scaffolds representing the draft genomes. The REAPR pipeline (8) was used to improve the assembly accuracy. Default parameters were used for all software unless otherwise noted.

This assembly procedure resulted in 210 scaffolds for *S. misionensis* strain ACT66 and 95 scaffolds for *S. albidoflavus* strain ACT77. Genome completeness and contamination were estimated using CheckM (9) in the lineage-specific mode. The estimated genome size for *S. misionensis* strain ACT66 is 8,312,220 bp, with a G+C content of 72.3% and an *N*_50_ value of 70,083 bp. The estimated genome size for *S. albidoflavus* strain ACT77 is 7,446,125 bp, with a G+C content of 73.2% and an *N*_50_ value of 1,152,190 bp. The genome completeness estimated by CheckM was 98.28% and 99.89%, and the contamination was 0.45% and 1.17% for *S. misionensis* strain ACT66 and *S. albidoflavus* strain ACT77, respectively, and they were classified as nearly complete with low contamination. We applied the method proposed by Parks and colleagues (10), which uses the software GTDBk and the Genome Taxonomy Database (GTDB; http://gtdb.ecogenomic.org) for assigning taxonomy to each assembled genome using the default parameters. Based on this software, our two isolates were classified as *S. misionensis* and *S. albidoflavus*.

Genome annotation was performed with PATRIC version 3.5.23 (11). It identified 7,936 coding DNA sequences (CDS) and 86 predicted noncoding RNAs (68 tRNAs and 18 rRNAs, encompassing 6 rRNA operons) for *S. misionensis* strain ACT66 and 6,819 CDS and 89 predicted noncoding RNAs (68 tRNAs and 21 rRNAs, encompassing 7 rRNA operons) for *S. albidoflavus* strain ACT77.

### Data availability.

This whole-genome shotgun project has been deposited at DDBJ/EMBL/GenBank under the accession numbers VOGW00000000 and VOGX00000000 for Streptomyces misionensis strain ACT66 and Streptomyces albidoflavus strain ACT77, respectively. The versions described in this paper are the first versions. Raw reads are available under the BioProject accession number PRJNA557451.
